# Reduced dose folinic acid rescue after rapid high-dose methotrexate clearance is not associated with increased toxicity in a pediatric cohort

**DOI:** 10.1007/s00520-021-06395-3

**Published:** 2021-07-08

**Authors:** Riitta Niinimäki, Henri Aarnivala, Joanna Banerjee, Tytti Pokka, Kaisa Vepsäläinen, Arja Harila-Saari

**Affiliations:** 1 Department of Children and Adolescents, Oulu University Hospital, Oulu, Finland; 2 PEDEGO Research Unit, University of Oulu, Oulu, Finland; 3 Children and Adolescents Hospital, Helsinki University Hospital, Helsinki, Finland; 4 Department of Pediatrics, Kuopio University Hospital, Kuopio, Finland; 5 Department of Women’s and Children’s Health, Uppsala University Hospital, Uppsala, Sweden

**Keywords:** Acute lymphoblastic leukemia, Methotrexate, Folinic acid, Toxicity

## Abstract

**Purpose:**

Low doses of folinic acid (FA) rescue after high-dose methotrexate (HD-MTX) have been associated with increased toxicity, whereas high doses may be related to a decreased antileukemic effect. The optimal dosage and duration of FA rescue remain controversial. This study was designed to investigate, whether a shorter duration of FA rescue in the setting of rapid HD-MTX clearance is associated with increased toxicity.

**Methods:**

We reviewed the files of 44 children receiving a total of 350 HD-MTX courses during treatment for acute lymphoblastic leukemia according to the NOPHO ALL-2000 protocol. Following a 5 g/m2 HD-MTX infusion, pharmacokinetically guided FA rescue commenced at hour 42. As per local guidelines, the patients received only one or two 15 mg/m^2^ doses of FA in the case of rapid MTX clearance (serum MTX ≤ 0.2 μmol/L at hour 42 or hour 48, respectively). Data on MTX clearance, FA dosing, inpatient time, and toxicities were collected.

**Results:**

Rapid MTX clearance was observed in 181 courses (51.7%). There was no difference in the steady-state MTX concentration, nephrotoxicity, hepatotoxicity, neutropenic fever, or neurotoxicity between courses followed by rapid MTX clearance and those without. One or two doses of FA after rapid MTX clearance resulted in a 7.8-h shorter inpatient time than if a minimum of three doses of FA would have been given.

**Conclusion:**

A pharmacokinetically guided FA rescue of one or two 15 mg/m^2^ doses of FA following HD-MTX courses with rapid MTX clearance results in a shorter hospitalization without an increase in toxic effects.

## Introduction

Methotrexate (MTX) is an antimetabolite that functions as a folic acid antagonist. MTX can be administered over a wide dose range, from 20 mg/m^2^ orally per week in maintenance chemotherapy for acute lymphoblastic leukemia (ALL) to a high intravenous dose of 33,000 mg/m^2^, when combined with folinic acid (FA) rescue [1]. High-dose methotrexate (HD-MTX) therapy given intravenously, defined as doses of 500 mg/m^2^ or higher, can cause significant toxicity [2]. To prevent toxicity, intravenous hydration with urine alkalinization and pharmacokinetically guided FA rescue is used in conjunction with HD-MTX therapy [2].

Despite supportive care, HD-MTX can induce nephrotoxicity, resulting in the accumulation of toxic concentrations of MTX, delayed elimination, and an increased risk of severe toxic adverse events [3]. Prolonged MTX exposure can cause myelosuppression, mucositis, hepatotoxicity, and, in severe cases, multi-organ failure [2]. MTX can also cause acute, subacute, and long-term neurotoxicity, which has been reported in 3.8 to 15.5% of ALL patients receiving HD-MTX [4, 5]. Increased toxicity after HD-MTX has been attributed to the use of inadequate FA doses and to a delay in commencing FA rescue [6]. However, the minimum duration and dose of FA rescue needed to prevent excess toxicity after HD-MTX remains unclear [7]. Furthermore, the use of higher doses of FA after HD-MTX has been associated with an increased risk of leukemic relapse [8, 9].

In the present study, we describe a series of 44 patients with pediatric ALL who received a total of 350 courses of 5 g/m^2^ HD-MTX followed by a pharmacokinetically guided FA rescue, including only one or two 15 mg/m^2^ doses in the case of rapid MTX clearance, with respect to clinical toxicity and length of hospital stay due to the HD-MTX therapy.

## Patients and methods

### Patients

Patients diagnosed with primary ALL between 2002 and 2007 and treated according to the standard risk or intermediate risk NOPHO ALL-2000 protocol in either Oulu or Kuopio University Hospital, Finland, were eligible for this study. We screened 50 eligible, consecutively treated ALL patients, excluding those with an early relapse during treatment (n = 3), an underlying syndrome (n = 2), and modified treatment due to severe toxicity caused by other antileukemic drugs (n = 1).

### Data collection

We retrospectively collected detailed treatment-related data (e.g., MTX concentrations, FA doses, dates and times, length of hospital stay) from medical records and reviewed and screened patient records for entries on neurotoxicity (posterior reversible encephalopathy syndrome, hypertensive encephalopathy, stroke-like syndrome, cerebrovascular complications, encephalitis), hospitalizations due to infection or mucositis, hepatotoxicity, and nephrotoxicity. Laboratory results were reviewed to assess hepatotoxicity and nephrotoxicity. We recorded the highest alanine aminotransferase value within 3 weeks from the start of the HD-MTX therapy, and creatinine levels obtained before and during intravenous hydration for HD-MTX. We assessed all brain imaging studies that had been performed during or after antileukemic therapy.

### High-dose methotrexate treatment and folinic acid rescue

The NOPHO ALL-2000 chemotherapy regimen included a total of eight courses of HD-MTX at 5 g/m^2^ with intrathecal MTX and FA rescue; further details of the study protocol have been published elsewhere [10]. According to the protocol, 10% of the HD-MTX dose was given during the first hour and the remaining 90% over the following 23 h. Intrathecal MTX was given just before the MTX infusion according to national practice. Intravenous hydration with 125 mL/m^2^/h, including 50 mmol/L of NaHCO_3_, was started at least 12 h before the commencement of the MTX infusion, according to national practice, and it was continued until serum MTX (S-MTX) had decreased to ≤ 0.2 μmol/L. Intravenous hydration was increased to 4500 mL/m^2^/24 h if S-MTX was ≥ 3 μmol/L at 36 h. Urine pH was measured at every voiding, and additional intravenous NaHCO_3_ was administered if the pH was < 7. S-MTX was monitored in all patients at 23, 36, 42, and 48 h from the onset of the MTX infusion and at least twice daily thereafter until S-MTX reached ≤ 0.2 μmol/L. S-MTX levels were measured with a fluorescence polarization immunoassay.

FA rescue with leucovorin commenced 42 h from the start of the MTX-infusion with a dose of 15 mg/m^2^. The local guidelines recommended 15 mg/m^2^ FA 42 h from the onset of the MTX infusion and every 6 h thereafter until the plasma MTX concentration is ≤ 0.2 μmol/L. Although the guidelines related to HD-MTX courses given as a part of NOPHO ALL-2000 therapy generally recommended at least three doses of FA regardless of MTX clearance speed, the local guidelines used in the hospitals participating in the present study gave no recommendation regarding the minimum number of FA doses. This resulted in only one or two FA doses being administered, if S-MTX had decreased to ≤ 0.2 μmol/L at 42 or 48 h. If the MTX concentration was ≥ 1 μmol/L at 42 h or later, the FA doses are adjusted, as shown in Table 1.

**Table 1:** Adjustment of leucovorin dose according to serum methotrexate (S-MTX)

A						
S-MTX at 42 h (μmol/L)	< 1.0	1–1.9	2–2.9	3–3.9	4–4.9	≥ 5.0
Extra leucovorin dose (mg/m^2^)	0	15	30	45	60	μmol/L S-MTX × kg bodyweight
B						
S-MTX at 48 h + (μmol/L)	< 1	1–1.9	2–2.9	3–3.9	4–4.9	≥ 5.0
leucovorin dose (mg/m^2^)	15	30	45	60	75	μmol/L S-MTX × kg bodyweight

^A^Additional leucovorin after S-MTX h42 is available, if S-MTX ≥ 1 μmol/L

^B^Calculation of leucovorin dose hour 48 and later (until S-MTX ≤ 0.2 μmol/L). The dose is calculated by the latest available S-MTX value

### Definitions

HD-MTX courses with S-MTX levels below 0.2 μmol/L by 48 h are referred to as rapid methotrexate clearance. Delayed MTX elimination was defined as an S-MTX concentration > 3 μmol/L at hour 36, and/or > 1 μmol/L at hour 42 or later, and/or > 0.2 μmol/L at hour > 72 [8, 11, 12]. Hyperfiltration was defined as a glomerular filtration rate ≥ 160 mL/min/1.73 m^2^ measured by ^51^Cr-EDTA clearance [13]. We graded the toxicities according to the Common Terminology Criteria for Adverse Events (CTCAE) version 4.0.

### Statistical analyses

Methotrexate concentrations between courses following one or two FA doses (courses with rapid MTX clearance; S-MTX ≤ 0.2 μmol/L at 42 or 48 h) were compared to courses following three or more FA doses using the Student’s *t* test and proportions between groups using the standard normal deviate (SND) test. The associations between the use of proton pump inhibitors (PPI) and MTX excretion and between FA doses and toxicities were analyzed with the Fisher’s exact test. Statistical analyses were performed with IBM SPSS Statistics for Windows, Version 26.0 (IBM Corp, Armonk, NY, USA) and StatsDirect Statistical Software, Version 3.2.8 (StatsDirect Ltd., England).

## Results

Forty-four patients (27 males) were included in the study, with a mean age of 5.7 years (range 1.3 to 15.9 years) at diagnosis. The patients received a total of 352 HD-MTX courses, but a total of 350 courses were analyzed, as information on two courses of HD-MTX administered at a non-participating hospital was missing. The mean (± SD) S-MTX concentrations were 52.2 (± 26.9) μmol/L at 23 h, 1.1 (± 2.6) μmol/L at 36 h, 0.40 (± 0.75) μmol/L at 42 h, and 0.26 (± 0.51) μmol/L at 48 h. A concentration of ≥ 10 μmol/L at 23 h was reached in 347/350 (99.1%) courses. Rapid MTX clearance was observed in 184 (52.6%) of the 350 courses, and S-MTX was ≤ 0.2 μmol/L already at 42 h in 58 (16.6%) of the 350 courses.

### Methotrexate clearance

**Fig. 1:**
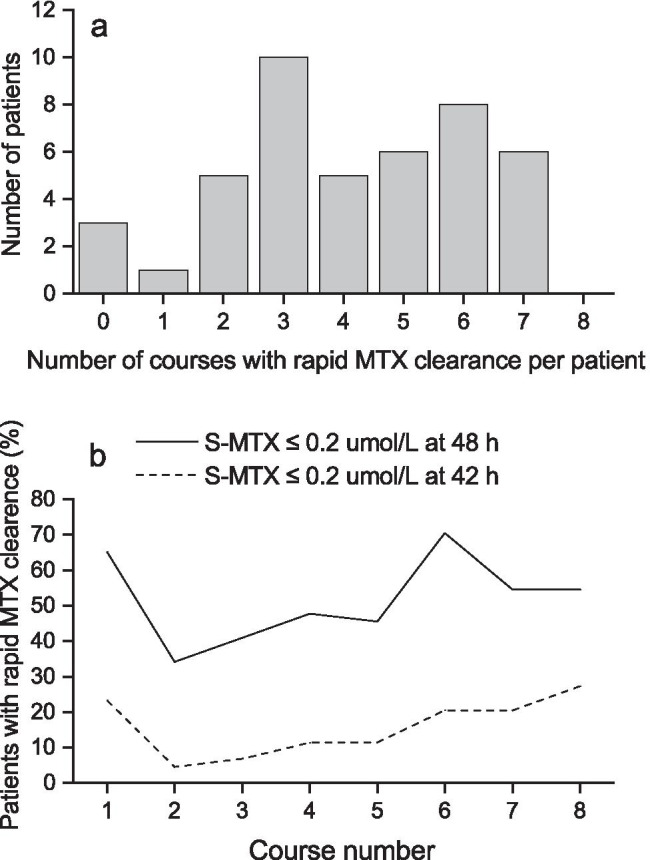
**a** Number of courses with rapid methotrexate clearance (serum methotrexate ≤ 0.2 μmol/L at hour 42 or hour 48) per patient. Data was missing for one course of high-dose methotrexate in two different patients. **b** Percentage of patients with rapid methotrexate clearance (serum methotrexate ≤ 0.2 μmol/L at hour 42 or hour 48) in different courses. Data was missing for one patient regarding the first course (1) of high-dose methotrexate and for one patient regarding the last course (8)

If the MTX concentration result was available within 6 h of sampling, patients with S-MTX levels ≤ 0.2 μmol/L at 42 h received only one dose of FA, while those with S-MTX levels ≤ 0.2 μmol/L at 48 h received two doses according to the protocol. Rapid MTX clearance resulting in only one or two FA doses being administered followed 181 courses of HD-MTX (51.7%). Only a single dose of FA was given following 55 HD-MTX courses (15.7%). Figure 1a shows the number of courses with rapid MTX clearance per child. An average of 4.1 (± 2.0) courses per patient was followed by rapid MTX clearance. There were only three patients without rapid MTX clearance in any course and no patient with rapid MTX clearance throughout all eight courses. Figure 1b shows the percentage of patients with rapid MTX clearance following each HD-MTX course.

The overall mean steady-state concentration at 23 h (throughout HD-MTX courses 1 to 8) did not differ significantly between courses with and without rapid MTX clearance (50.5 vs. 54.1 μmol/L, *p* = 0.213). However, the mean 23-h steady-state concentration differed between courses with rapid and non-rapid MTX clearance in HD-MTX courses 1 (51.7 vs 71.2 μmol/L, *p* < 0.05), 2 (42.7 vs 62.9 μmol/L, *p* < 0.01), and 6 (46.7 vs 33.4 μmol/L, *p* < 0.05). The 23-h steady-state concentration was not affected by age or gender. Delayed elimination was observed in 15 courses (4.3%).

### Toxicity

Fewer FA doses were not associated with an increased risk of clinical toxicity. None of the patients in the study presented signs of acute or subacute clinical neurotoxicity according to the patient records. Brain magnetic resonance imaging (MRI) scans were routinely performed at the cessation of ALL therapy in Oulu, and of a total of 22 patients with brain MRI scans available, no leukoencephalopathy or other potential signs of neurotoxicity were detected.

There was no statistically significant difference in infections, as 7.3% (12/165) of the courses with rapid MTX clearance and 8.7% (14/161) without rapid MTX clearance were followed by intravenous antibiotic treatment within 3 weeks of HD-MTX therapy (*p* = 0.282); data was missing for 24 courses. Only 1.5% of MTX courses were followed by grade III–IV mucositis requiring hospitalization, but the data regarding mucositis was not always been reliably documented; however, four cases occurred after a course without and one occurred after a course with rapid MTX clearance (*p* = 0.112).

Three cases of grade IV alanine aminotransferase elevation were observed, one with normal and two with rapid clearance (*p* = 0.445), within 3 weeks after HD-MTX, but the difference was not statistically significant.

Sufficient data to evaluate nephrotoxicity were available for 302 courses, and nephrotoxicity occurred in 5/151 (3.3%) courses with and 9/151 (6.0%) courses without rapid MTX clearance (*p* = 0.417), which were all grade II with the exception of one grade III toxicity associated with severely delayed MTX elimination. Nephrotoxicity was significantly more common in courses with delayed elimination: 4/14 (28.5%) of courses with delayed elimination, and 10/288 (3.4%) without delayed elimination were accompanied by nephrotoxicity (*p* < 0.001).

A subanalysis comparing the courses followed by only one dose of FA with courses followed by two or more doses of FA was also performed, but it revealed no differences in the incidence of toxic effects, either.

### Concomitant medication

Nitrous oxide was not used for sedation during any of the courses. PPIs were administered during 82 out of 184 (44.6%) courses with rapid clearance and during 87 out of the remaining 166 (52.4%) courses (*p* = 0.113). Delayed elimination was observed after 9/169 (5.3%) courses during which PPIs were administered and 6/181 (3.3%) courses during which PPIs were not administered; the difference was not statistically significant (*p* = 0.360).

### Hyperfiltration

The glomerular filtration rate was measured before 52 HD-MTX courses by ^51^Cr-EDTA clearance, and hyperfiltration was observed preceding 21/52 courses (40.3%). There were no differences in MTX steady-state concentrations or clearance, whether or not hyperfiltration was observed before the HD-MTX course.

### Length of hospital stay

The mean length of hospital stay due to HD-MTX therapy, from the start of intravenous hydration to the last dose of FA, was 68.3 h (± 2.8 h) in the whole cohort. In courses with rapid MTX clearance, the mean hospital stay was 53.0 h (± 1.7 h). If an FA rescue regimen of at least three doses had been used regardless of MTX clearance speed, the overall mean hospital stay would have increased to 72.4 h in the whole cohort and to 60.8 h in courses with rapid MTX clearance. Hence, the shorter FA rescue following rapid MTX clearance resulted in the shortening of the hospital stay by 4.1 h in the whole cohort (*p* < 0.001) and by 7.8 h in courses with rapid clearance (*p* < 0.001).

### Event-free and overall survival

Five-year event-free survival (EFS) and overall survival (OS) in the cohort were 89% and 97%, respectively, which were consistent with the results of the NOPHO ALL-2000 protocol in general, even considering the exclusion of the three early relapses from the present cohort [10].

## Discussion

This study is the first report of ALL patients receiving only one or two doses of FA after a 5 g/m^2^ HD-MTX infusion. The results suggest that in the context of rapid MTX clearance, a shorter FA rescue may be sufficient. Although guidelines related to HD-MTX courses given as a part of NOPHO ALL-2000 therapy generally recommended at least three doses of FA regardless of MTX clearance speed, the local guidelines used in the hospitals participating in the present study provided no recommendation regarding the minimum number of FA doses. This resulted in administering only one or two FA doses in the case of rapid MTX clearance. Following 181 of 350 5 g/m^2^ HD-MTX courses, only one or two doses of FA were administered due to rapid MTX clearance. In total, 41 out of 44 patients exhibited rapid MTX clearance following at least one course of HD-MTX. A shorter FA rescue following rapid MTX clearance was associated with a shorter hospital stay without increased toxicity. Severe toxicities were uncommon and did not differ between the courses with or without rapid MTX clearance.

All patients received extended hydration with 3000 mL/m^2^/24 h of fluids including 50 mmol/L NaHCO_3_ over 12 h, according to the national practice, with extra NaHCO_3_ given if urine pH was < 7. Urine alkalinization has been shown to be the most important factor influencing MTX clearance, and the active alkalinization may partially explain the rapid clearance observed in the present cohort [2]. Extended hydration is deemed important, although a Danish randomized study by Mikkelsen et al. found no difference between 4- and 12-h prehydration with urine alkalinization [14, 15]. However, the study reported markedly higher MTX concentrations after HD-MTX 5 g/m^2^ at hours 23, 36, and 42 compared to the present study as well as a remarkably higher incidence of delayed elimination and nephrotoxicity: 47% and 18.5%, compared to 4.3% and 3.3% in the present study, respectively. The results of these two studies including patients treated with the same chemotherapy regimen are drastically different, implying a role of other unrecognized factors beyond extended hydration and urine alkalinization—such factors could include ethnic differences in genetic polymorphisms in the metabolic and cellular transport pathway of methotrexate, concomitant medication, or differences in other local practices. These differences call for a further study on the effect of extended prehydration in a population different than in Mikkelsen et al. [14].

FA is used to neutralize the effect of MTX on healthy cells after prolonged exposure. It has been reported to be especially effective against mucous membrane and gastrointestinal toxicity, hematological toxicity, and neurotoxicity. Elevated MTX-to-FA ratios at 42 h reportedly increase the risk for leukoencephalopathy [4]. Cohen suggested that neurotoxicity observed after HD-MTX is mainly due to inadequate FA rescue and that FA rescue should commence 24–36 h after MTX exposure [6]. Cohen also stated that higher doses of FA could explain the reduced incidence of neurotoxicity in some protocols [16]. Our study suggests that the pharmacokinetics of MTX following HD-MTX therapy may play a more important role regarding the subsequent toxic effects than the starting time or dosing of FA rescue. The finding of HD-MTX courses with rapid MTX clearance followed by only one or two doses of FA started 42 h from the initiation of HD-MTX infusion being associated with acceptable toxicity might also be explained by spontaneous recovery of folate metabolism, which has been observed by Frickel et al., who reported spontaneously increased levels of 5-methyltetrahydrofolate 42 h after a 24-h infusion of 5 g/m^2^ HD-MTX [17].

There were no clinically significant differences between mucositis, neutropenic fever, nephrotoxicity, or hepatotoxicity between the rapid and non-rapid MTX clearance groups. As blood sampling varied largely between patients, we could not analyze hematological toxicity. No cases of acute or subacute neurotoxicity occurred in the study cohort. Brain MRI revealed no signs of leukoencephalopathy in 22 of the 44 patients undergoing a screening MRI at the end of ALL therapy. However, neuropsychological studies were not routinely performed on all of the patients, and thus, some milder neuropsychological changes may have gone unnoticed. The incidence of mucositis is underestimated, as only the cases of mucositis leading to hospitalization were recorded, and it is likely that a significant proportion of the patients hospitalized due to neutropenic fever also had some degree of mucositis, which had not been documented in the files. However, the rates of both mucositis and infection were lower than in the previously mentioned study by Mikkelsen et al., who reported mucositis in 9% of their cohort [14]. In our cohort, the overall incidence of nephrotoxicity of 4.6% falls in the lower part of the 2–12% range reported in the literature [1]. Three patients developed grade IV hepatotoxicity, with two following rapid MTX clearance. A transient increase in transaminases is common after HD-MTX and is usually not regarded as clinically significant [18]. During the NOPHO ALL-2000 protocol, prior to awareness of the potential interaction, PPIs were commonly used during HD-MTX infusions. Although numerous reports of PPIs increasing the risk of delayed clearance have been published, the use of PPIs did not differ between the clearance groups and did not appear to be associated with delayed elimination in the present study [19].

Glomerular hyperfiltration was common in the subgroup of patients with the result of glomerular filtration rate measurement available, which is in accordance with earlier reports on children with cancer [13]. However, hyperfiltration was equally common in both clearance groups in the present cohort.

There have been concerns that the carry-over effect of FA could decrease the antileukemic effect of HD-MTX therapy. According to Sterba and colleagues, high pretreatment folate concentrations were associated with lower MTX exposure when measured with homocysteine accumulation [20]. In an earlier Nordic study, higher FA doses were associated with a higher risk of relapse [8]. On the other hand, courses with rapid MTX clearance might confer less antileukemic effect due to shortened exposure to MTX. Indeed, in other protocols, shortening HD-MTX infusion times has been shown to increase the risk of relapse [21]. The importance of lowering and postponing the FA dose is still unclear, but it is tempting to assume that a shorter and lower FA rescue had a positive impact on the overall antileukemic effectiveness of the therapy in the present cohort, considering that the EFS and OS were excellent, although the sample size was small and the three early relapses were excluded from the study. Nevertheless, in light of the present study, a reduced FA rescue appears adequate in the context of rapid MTX clearance after HD-MTX infusion. Determining whether extended prehydration plays a role in the rapid MTX clearance and the low incidence of delayed elimination in the present population requires further study.

## Conclusion

A pharmacokinetically guided FA rescue of one or two 15 mg/m^2^ doses of FA following HD-MTX courses with rapid MTX clearance results in a shorter hospitalization duration without an increase in toxic effects.

## Data Availability

The data that support the findings of this study are available from the corresponding author upon reasonable request.

## Abbreviations

ALL: Acute lymphoblastic leukemia
CTCAE: Common Terminology Criteria for Adverse Events
EFS: Event-free survival
FA: Folinic acid
HD-MTX: High-dose methotrexate
MRI: Magnetic resonance imaging
MTX: Methotrexate
OS: Overall survival
PPI: Proton pump inhibitor
S-MTX: Serum methotrexate
